# Optimal bi-planar gradient coil configurations for diamond nitrogen-vacancy based diffusion-weighted NMR experiments

**DOI:** 10.1007/s10334-023-01111-0

**Published:** 2023-08-14

**Authors:** Philipp Amrein, Fleming Bruckmaier, Feng Jia, Dominik B. Bucher, Maxim Zaitsev, Sebastian Littin

**Affiliations:** 1 Division of Medical Physics, Department of Diagnostic and Interventional Radiology, University Medical Center Freiburg, Faculty of Medicine, University of Freiburg, Killianstrasse 5a, 79106 Freiburg, Germany; 2 Department of Chemistry, TUM School of Natural Sciences, Technical University of Munich, 85748 Garching, Germany

**Keywords:** Magnetic resonance, MRI, Gradient coils, Diffusion weighted imaging, NV–NMR, NV centers, Quantum Sensing, Diamonds

## Abstract

**Introduction:**

Diffusion weighting in optically detected magnetic resonance experiments involving diamond nitrogen-vacancy (NV) centers can provide valuable microstructural information. Bi-planar gradient coils employed for diffusion weighting afford excellent spatial access, essential for integrating the NV-NMR components. Nevertheless, owing to the polar tilt of roughly $55^{\circ }$ of the diamond NV center, the primary magnetic field direction must be taken into account accordingly.

**Methods:**

To determine the most effective bi-planar gradient coil configurations, we conducted an investigation into the impact of various factors, including the square side length, surface separation, and surface orientation. This was accomplished by generating over 500 bi-planar surface configurations using automated methods.

**Results:**

We successfully generated and evaluated coil layouts in terms of sensitivity and field accuracy. Interestingly, inclined bi-planar orientations close to the NV–NMR setup’s requirement, showed higher sensitivity for the transverse gradient channels than horizontal or vertical orientations. We fabricated a suitable solution as a three-channel bi-planar double-layered PCB system and experimentally validated the sensitivities at $28.7 \mathrm mT/m/A$ and $26.8 \mathrm mT/m/A$ for the transverse $G_{x}$ and $G_{y}$ gradients, and $26 \mathrm mT/m/A$ for the $G_{z}$ gradient.

**Discussion:**

We found that the chosen relative bi-planar tilt of $35^{\circ }$ represents a reasonable compromise in terms of overall performance and allows for easier coil implementation with a straight, horizontal alignment within the overall experimental setup.

## Introduction

Nitrogen-vacancy (NV) based nuclear magnetic resonance (NV–NMR) in diamonds is a promising approach for investigating the biological micro- to nanoscale domain, such as single cells [1]. NV-based magnetometry provides broadband detection of magnetic fields with high sensitivity and small sample sizes [2–4]. In optically detected NV–NMR, the sample’s magnetization is detected by measuring the fluorescence of nearby NV centers [5, 6].

NMR-based spectroscopy [7] and imaging (MRI) [8] are, on the other hand, well-established measurement techniques, extensively used for medical and technical applications. Nuclear magnetization is measured by detecting oscillating magnetic fields precessing around a static external magnetic field [9].

In NMR/MRI of sub-millimeter samples, the signal-to-noise ratio (SNR) is dominated by the resistive noise of the MR receiver coil [10]. By replacing the MR receiver coil with an NV-diamond detector, the SNR can potentially improve for positions close to the NV centers [11, 12].

Within the microscopic scale of the diamond’s area of interest [6], particle motion occurs through diffusion. By investigating these diffusion properties, it is possible to gain insights into the microstructural level. [13, 14]. Technically, diffusion-weighted NMR/MRI studies the diffusion properties of a sample by phase accumulation of the NMR signal [15]. This is achieved as signal preparation by adding spatially varying gradient fields, which are generated by corresponding gradient coils. To capture the full directional information of diffusion, three orthogonal gradient fields are necessary [13].

In MRI, gradient coils are generally used to induce spatially dependent frequency variations of the MR signal, providing spatial information [16, 17]. In most standard cases, gradient fields $G_{i}$ are linear variations of the magnetic field component along the main static magnetic field: $G_{i}=\frac{\partial B_{z}}{\partial \vec {x}_{i}}$. The static field, called $B_{0}$, is conventionally aligned here along the z-axis of the coordinate system. For full 3D encoding, three orthogonal gradient fields are required to define the internal MR coordinate system (x, y, z). Each gradient field is generated by a separate gradient coil, commonly named after the direction of the induced field variation within the given coordinate system, i.e., ”$G_{x}$”, ”$G_{y}$”, and ”$G_{z}$”.

**Fig. 1:**
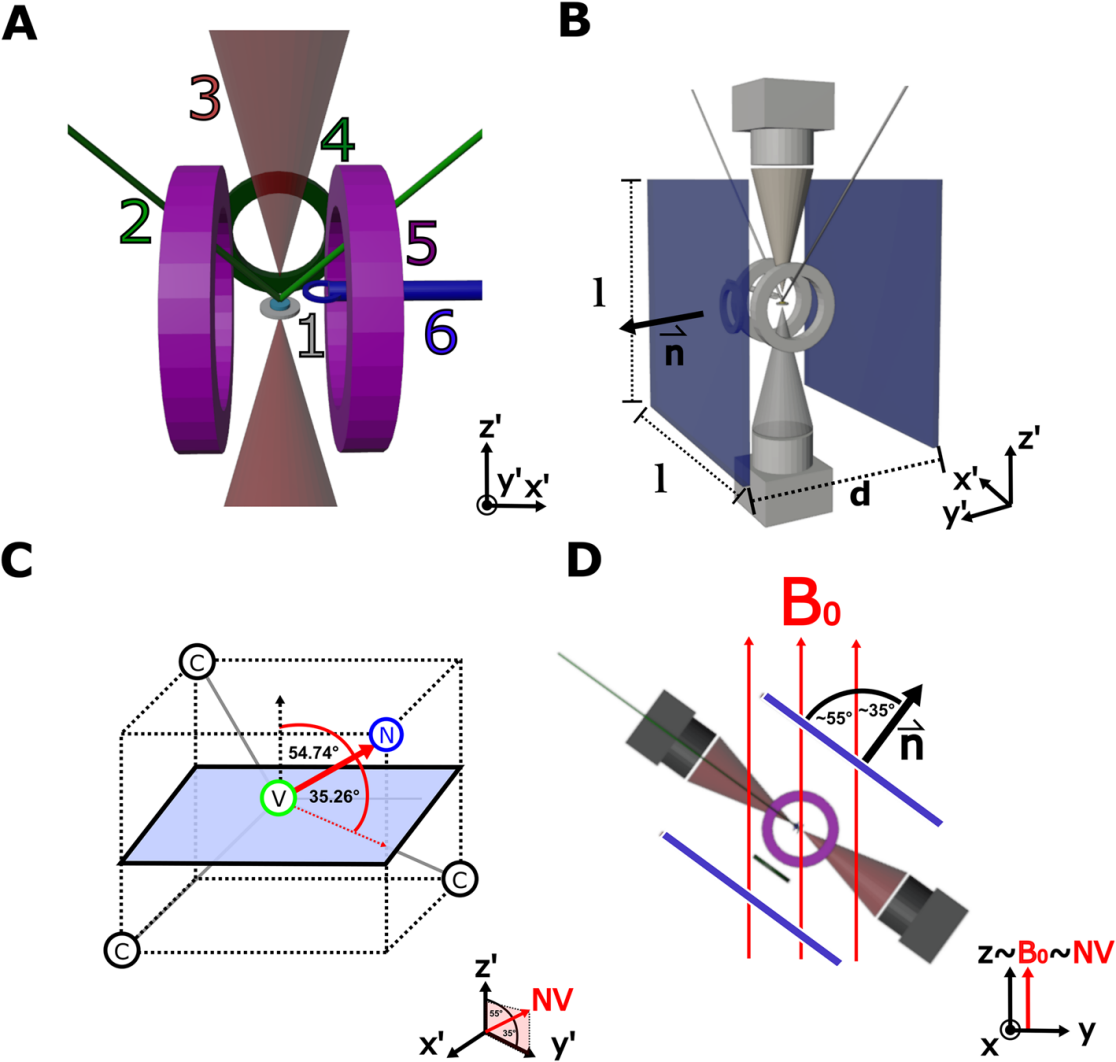
**A:** Simplified scheme of the given NV-NMR setup[18]: 1. NV-Diamond and microfluidic assembly mounted directly on its surface, 2: Green excitation laser, 3: Red emitted photons and receive photo diodes, 4: RF Calibration Coil, 5: RF Excitation Coil, 6: Microwave Coil to drive the NV-center; **B:** Integration of a bi-planar gradient surface (shown in blue) within the overall setup (shown in grey). The configuration shown is the variant that was finally built. The optimized bi-planar parameters (square side length *l*, surface separation *d*, normal orientation $\vec {n}$) are indicated. **C:** Diamond lattice cell with (100) plane (shown in light blue) and [111] NV center (red arrow)[6]. Note the angle of $54.74^{\circ }$ between the NV symmetry axis and the diamonds surface normal. **D:** Relative orientation of the main $B_{0}$ field within the experimental NV-NMR setup. The $B_{0}$ field has to match the NV symmetry axis. In the MR coordinate system, the $B_{0}$ is per convention in direction of the z-axis

In the context of NV-NMR, the necessary orientation of the static $B_{0}$ field within an NV-NMR is defined by the NV center (refer to Fig. 1C, D). For our experimental setup (described separately in [18]), we utilize NV centers solely along the [111] direction. Throughout the NV-NMR experiment, the diamond is oriented such that its (100) surface normal aligns with the laboratory’s vertical z-axis. The projection of the [111] NV-axes onto the xy-plane is furthermore parallel to the y-axis. This orientation is determined by constraints of the experimental setup such as the microfluidic system and the lasers incident on the diamond surface. For such orientation, NV-centers in [111] direction are now positioned at an angle of $54.74^{\circ }$ ($\approx 55^{\circ }$) with respect to both the laboratory z-axis and the diamond’s (100) surface[19].

The NV–NMR signal relies on the Zeeman splitting of electronic NV states [20], where the NV axis typically aligns with the external $B_{0}$ field. In our case, to align with the NV quantization axis, the $B_{0}$ field must also be oriented approximately $55^{\circ }$ relative to the diamond’s (100) surface normal (z-axis). Now, if we define our set of NV-specific gradient axes, we rotate our coordinate system by $55^{\circ }$ around the x-axis to achieve a $G_{z}$ parallel to $B_{0}$. Consequently, in comparison to the laboratory coordinates, $G_{x}$ remains along the x-axis, while $G_{y}$ and $G_{z}$ undergo rotation by $55^{\circ }$. This angulation requirement causes a significant difference from the regular orientation of the main and gradient fields relative to the laboratory coordinate system and must be taken into account.

This study focuses on incorporating a three-channel gradient system for diffusion weighting and potentially future imaging applications into an NV-NMR experimental setup, aimed at investigating diffusion properties in microfluidic systems [21]. The NV-NMR setup and diffusion-based experiments are presented separately in detail by a separate publication by F. Bruckmaier et al. [18].

**Fig. 2:**
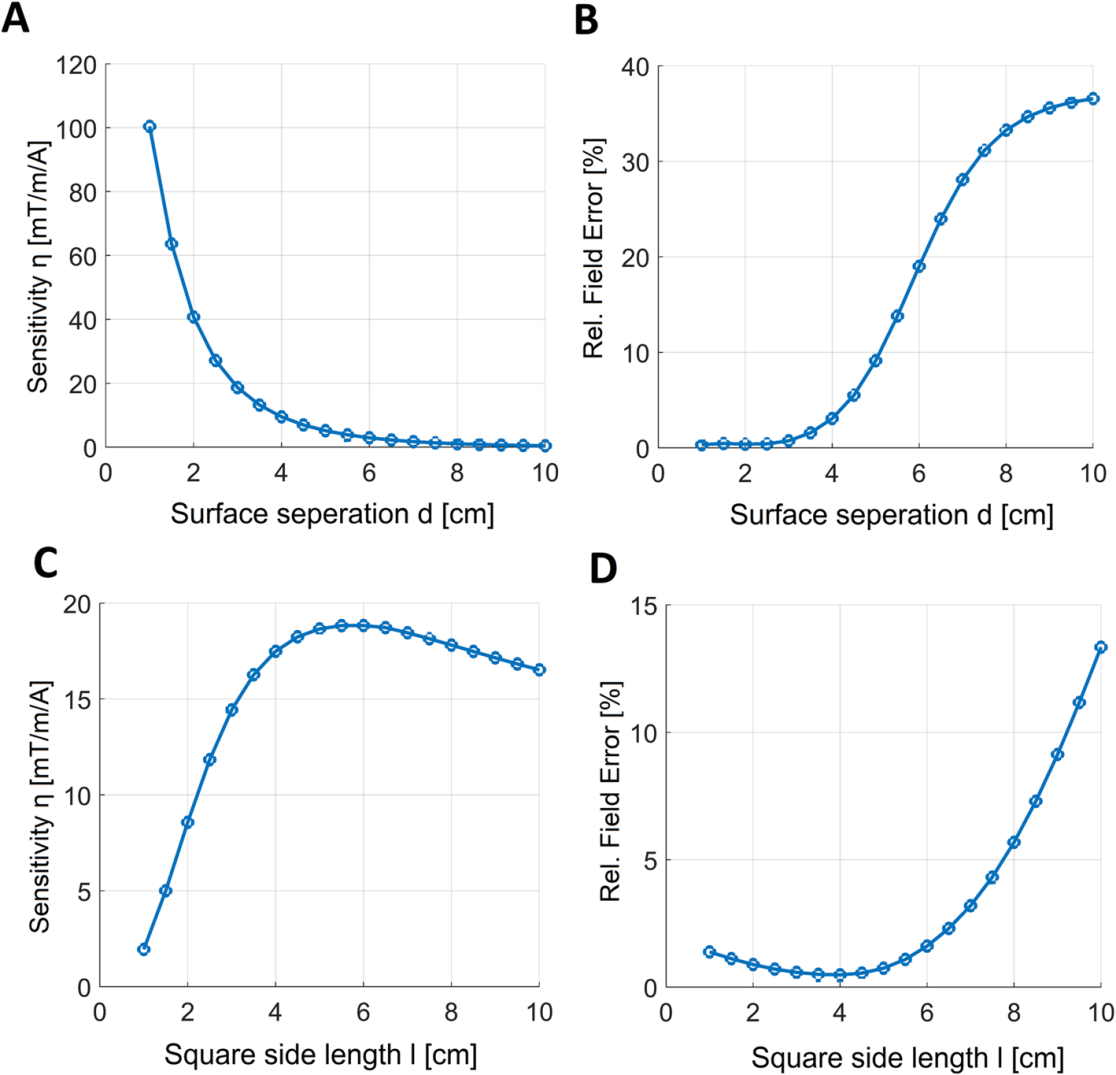
Simulated gradient sensitivities $\eta =G/I$ and relative field errors for different surface separations *d* and square side lengths *l* of the bi-planar gradient geometry. The plots show results for the $G_{x}$ channel with bi-planar surface orientation identical to the one shown in Fig. 1B. **A,B** Sensitivity ($\eta$ in $mT/m/A$) and relative field error in % versus bi-planar separation *d* with *d* from 1*cm* up to 10*cm*. The square side length is kept constant to $l=5cm$. **C,D** Resulting sensitivity ($\eta$ in $mT/m/A$) and relative field error in % versus different square side lengths *l* from 1*cm* up to 10*cm* for a fixed separation of $d=3cm$. The sensitivity reduces considerably as the surface separation *d* increases. For separations greater than $d=3cm$, the field error begins to rise. With regards to the square side length *l*, the sensitivity increases as the surfaces become larger, up to $l=5cm$. Surprisingly, the gradient performance (i.e. high sensitivity, low error) starts to significantly deteriorate for larger surfaces with $l>5cm$. We attribute this reduction in performance for larger surfaces to the decrease in mesh resolution in the central region. It should be noted that the shown sensitivities of the final layouts in Fig. 5 differ by a factor of about two because the PCB coils were constructed in double layers, which doubles the sensitivity. Additionally, the sensitivity has changed to some extent since the the number of wire turns were adjusted, which was necessary to avoid overlapping tracks

Due to the constraints and requirements of the available NV-NMR setup, we choose a bi-planar design with square current-carrying surfaces (See Fig. 1B). On a much larger scale, bi-planar gradient coils are already established for open whole-body MRI systems [22–26]. Since the distance between the gradient coils to be designed and the $B_{0}$ magnet used in this experiment will be relatively large, no active shielding layers are included. We pay particular attention to finding optimal square side lengths, surface separations, and orientations of bi-planar coil configurations.

Furthermore, this work presents a general investigation of the gradient performance of a bi-planar system as a function of geometrical parameters, specifically the relative orientation of the coil with respect to the main magnetic field $B_{0}$. Apart from existing work on cylindrical coils [27] that quantitatively relate surface geometry to performance, the authors are not aware of similar work on bi-planar configurations.

**Fig. 3:**
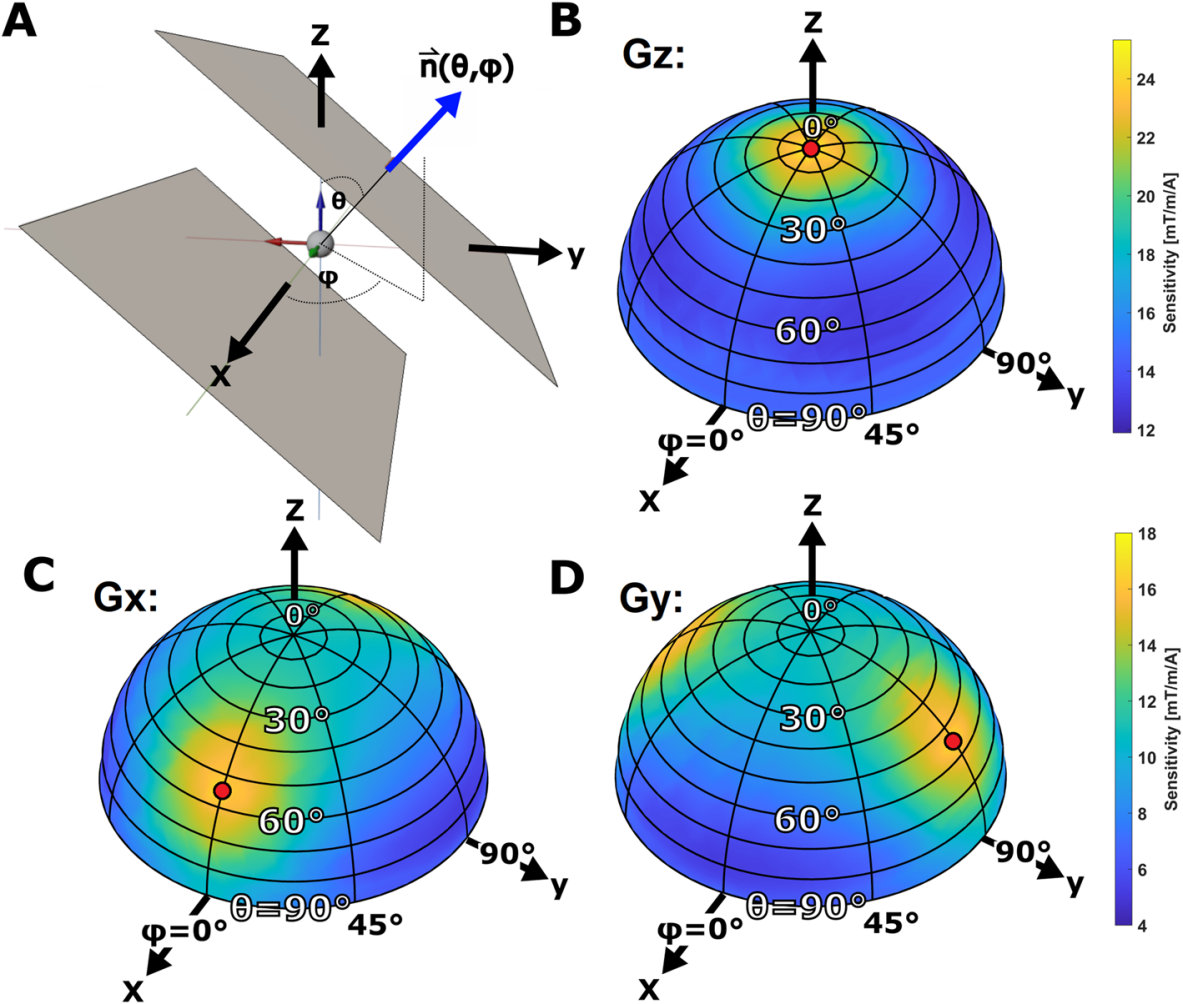
Simulated gradient sensitivities $\eta =G/I$ as a function of surface orientation $\vec {n}(\theta ,\phi )$: **A:** Definition of the normal orientation of the bi-planar current-carrying surface with the polar angle $\theta$ and the azimuthal angle $\phi$. The NMR/MRI coordinate convention is used, i.e., the z-axis coincides with the direction of the main field $B_{0}$. **B-D:** Simulated sensitivities for the $G_{x}$, $G_{y}$, and $G_{z}$ gradient coils as a function of normal orientation distributed on the hemisphere. The two other bi-planar parameters are set constant to $l=5cm$ and $d=3cm$. The sampled normals are densely and regularly distributed on the hemisphere with more than 100 values. For better illustration, the data points are linearly interpolated to create a smooth surface. For the $G_{z}$-channel, the solution with the highest sensitivity (red dot) of approximately $25 mT/A /m$ has a polar angle of $0^{\circ }$. For the transverse $G_{x}$ and $G_{y}$-channels, optimal polar angles of approximately $55^{\circ }$ were found with respective azimuth values of $0^{\circ }$ for the $G_{x}$ and $90^{\circ }$ for the $G_{y}$ channel. Again, it should be noted that the shown sensitivities differ from the final layouts in Fig. 5 because we built the final PCB coils in double layer configuration and we adjusted the number of wire turns to avoid overlapping tracks

## Theory and methods

### Integration of a gradient system into the NV–NMR setup

Integrating a gradient system into the existing NV-NMR experimental setup presents challenges due to the limited space available, which is primarily occupied by NV-NMR components [18]. The goal is to identify a suitable surface geometry that accommodates these restrictions.

The original NV-NMR experimental setup is situated within a custom-made $0.175T$ magnet (3T-215-RT, Superconducting Systems INC., Billerica, USA) and primarily comprises an NV-doped diamond, a microfluidic sample directly mounted on the diamond’s surface, an excitation laser beam, and a photodiode for fluorescence readout [18]. Additionally, a microwave coil, positioned close to the diamond, is employed to drive the spin states of the NV centers. A schematic of this setup is depicted in Fig. 1A. Unobstructed optical paths are required for both the excitation laser and fluorescence readout, which must not be blocked by the gradient system.

Owing to these spatial constraints, implementing the widely-used cylindrical gradient coil geometry proves difficult. We have also opted against employing the mono-planar approach, as it typically yields poor gradient homogeneity in the direction perpendicular to the current-carrying surface [28]. Consequently, this study examines bi-planar coil configurations with two parallel square surfaces.

In accordance with the NMR/MRI coordinate convention, the three orthogonal gradient fields $G_x$, $G_y$, and $G_z$, are aligned with the *x*, *y*, and *z* axes of the internal NMR/MRI coordinate system. The static $B_{0}$ field is also aligned along the z-coordinate axis.

**Fig. 4:**
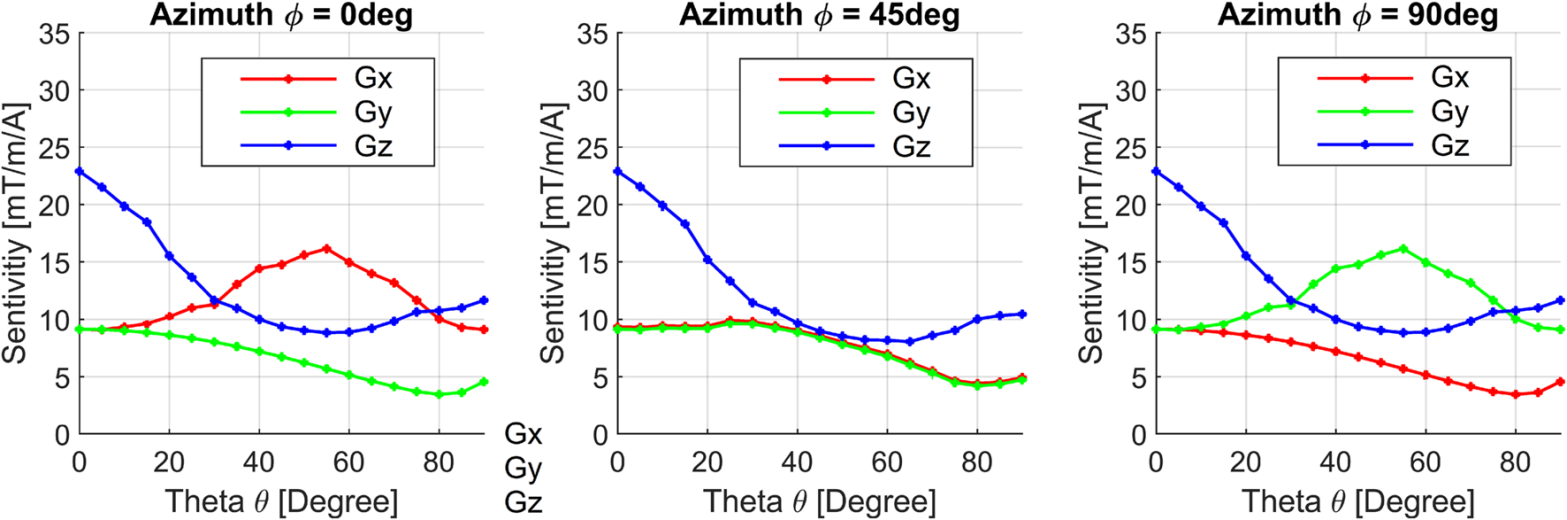
Simulated gradient sensitivities $\eta =G/I$ as a function of surface orientation $\vec {n}(\theta ,\phi )$. Same underlying data as shown in Fig. 3, but reduced for better visualization to azimuthal angles of $0^{\circ }, 45^{\circ }$ and $90^{\circ }$. For the $G_{z}$-channel (in blue) the solution with highest sensitivity is in $''z''$ direction with a polar angle of $0^{\circ }$. For the $G_{x}$ and $G_{y}$ channel an optimal polar angles of $~55^{\circ }$ were found with respective azimuth values of $0^{\circ }$ for $G_{x}$ and $90^{\circ }$ for $G_{y}$

**Fig. 5:**
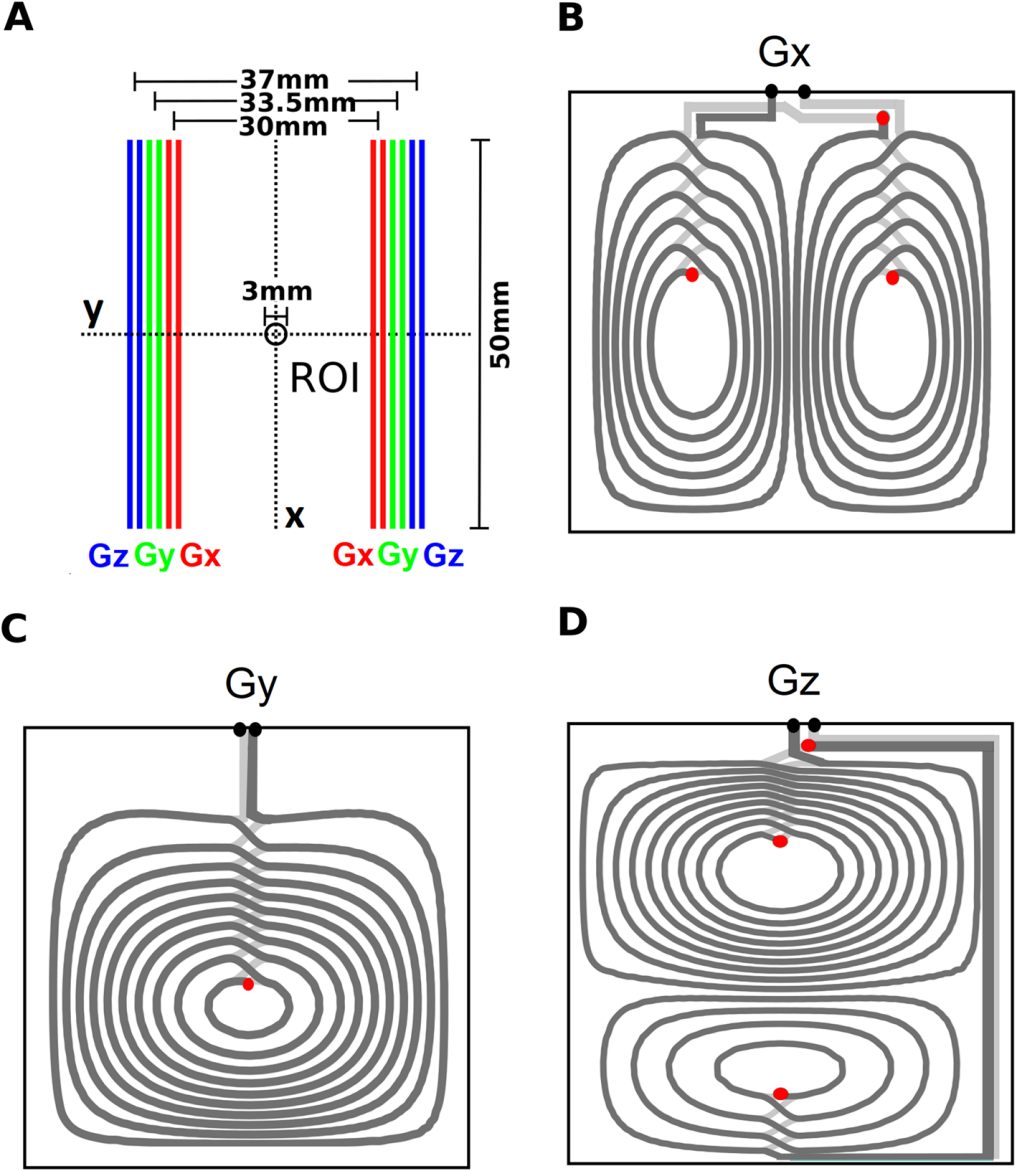
Technical details and PCB layouts of the built bi-planar gradient system: **A:** Spatial position of the different gradient PCBs carrying the coil layouts. **B-D:** Wire patterns of the fabricated gradient system for the $G_{x}$, $G_{y}$ and $G_{z}$ channel. The square side length for the shown layout are chosen for all channels to be 5*cm* wheres the bi-planar surface separations are chosen to be 3*cm*, 3.35*cm* and 3.75*cm*. The current-carrying surface orientation is chosen as an polar tilt of $35^{\circ }$ and a azimuth of $90^{\circ }$ in y-direction, which allows for an upright, horizontal orientation in the laboratory coordinate system. The tracks of the individual PCBs were generated with a track width of 0.5*mm*. The copper thickness of the used double layered PCB is $35\mu m$. Each of the two boards of the three bi-planar gradient channels carries the conductors over two layers. The two layers allow a spiral back-and-forth (dark grey, light grey) connection without additional feed lines. The conductive connection between the upper and lower layers are indicated by the red dots. The final ports are shown by black dots. The black frame indicates the boundary of the PCBs

For the NV–NMR setup, the $B_{0}$ field must be inclined at a $55^{\circ }$ angle relative to the NV-diamond crystal and its (100) surface plane to align with the orientation of the NV quantization axis (see Fig. 1D). However, since our focus here is solely on the gradient system design, we maintain the NMR/MRI convention, i.e., with a $B_{0}$ field and an NV quantization axis aligned along the z-axis. We can account for the resulting coil orientations in the original NV-NMR setup by rotating the bi-planar surface back by the same polar angle of $55^{\circ }$ (see Fig. 1D).

### Search for the optimal bi-planar geometry

To identify the optimal bi-planar gradient geometry for the NV-NMR setup described earlier (see Fig. 1), we utilized the MATLAB-based open-source design tool, *CoilGen* [29] (https://github.com/Philipp-MR/CoilGen). CoilGen allows for the rapid automatic generation of numerous coil configurations by sampling various geometric parameters of the bi-planar surface. By analyzing the performance of the resulting coil variants, we can determine the optimal configurations. We investigated the following geometric parameters of the bi-planar surface:

1. Planar surface separation *d* (ranging from 10 mm to 200 mm)
2. Square side length *l* (ranging from 10 mm to 200 mm)
3. Surface normal orientation $\vec {n}$ relative to $B_{0}$

For understanding, a full grid search across these three variables involves testing each variable across approximately 10-20 distinct values, results in several thousand combinations. However, due to the geometric limitations of the NV setup, not all combinations derived from the grid search are feasible for integration into the experiment. To narrow the scope of this paper, we choose to vary only one parameter while maintaining fixed at practical and reasonable values for the others. This approach allows us to present qualitative trends that emphasize the impact of geometric parameters on sensitivity.

The target region for the gradient system is defined as a spherical volume with a diameter of 3 mm at the center of the diamond, which has dimensions of 2 mm $\times$ 2 mm $\times$ 0.5 mm, corresponding to the geometric center of the evaluated bi-planar surfaces. The mesh resolution for the bi-planar surface is set to 2178 regularly spaced mesh nodes, representing a compromise between discretization accuracy and computation time, considering that multiple cases will be computed. Although the number of nodes could have been further optimized, we did not address this aspect in the present work to maintain a specific focus.

The value range investigated for the square side length *l* was chosen as 10 mm to 200 mm, and for the bi-planar separation *d* as 10 mm to 200 mm, based on the design constraints imposed by the NV-NMR components.

**Fig. 6:**
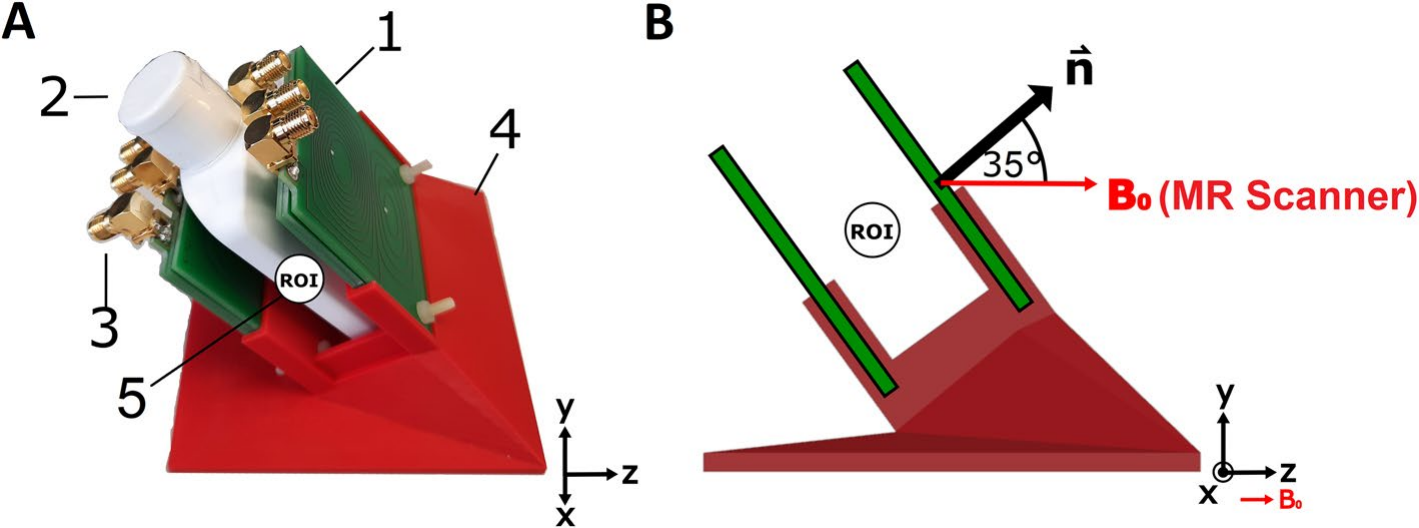
**A** Photograph of the experimental setup for the field measurement of the realized gradient system. Components: 1: PCBs with the printed wire patterns, 2: phantom bottle, 3: SMA connector, 4: 3D-Printed (PLA) angulated coil holder, 5: Region of interest with diameter of 3 mm **B** Side view of the setup: The tilt of the printed coil holder(4) allows for the z-direction of the gradient PCBs to match the $B_{0}$ direction of the clinical MR scanner

**Fig. 7:**
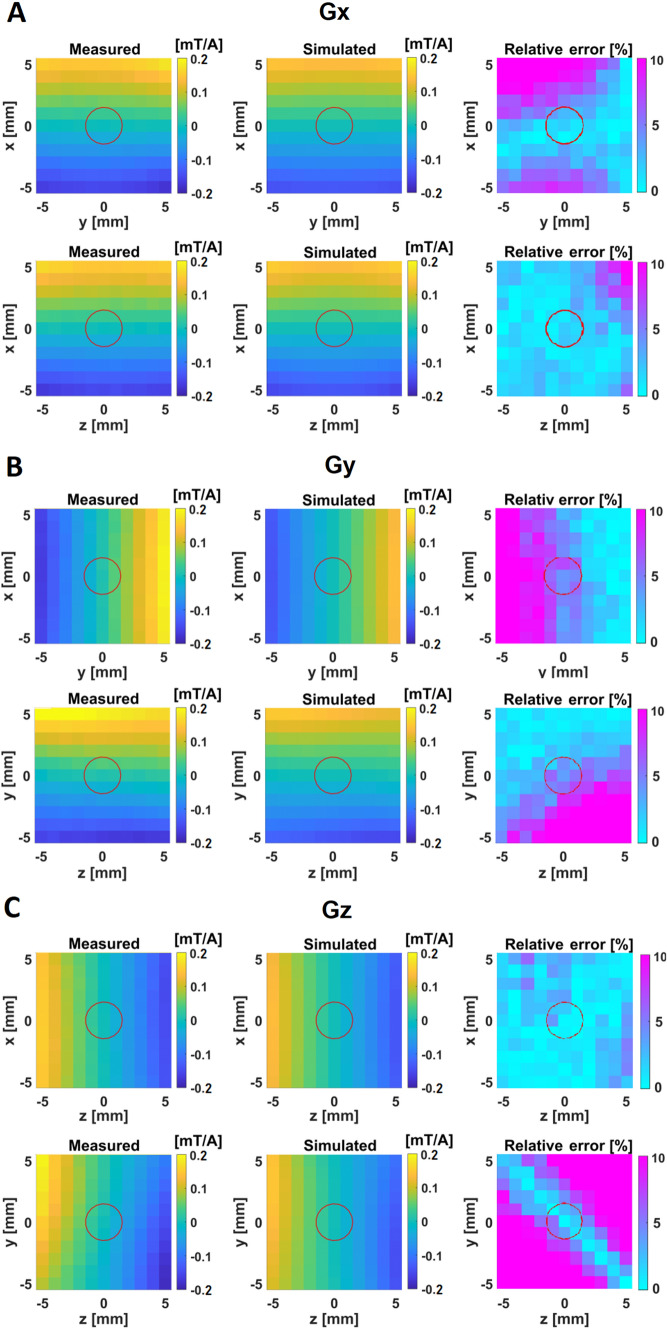
Measured and simulated gradients field for the individual channels of the built system. Plots of shown in different slices of the target region: **A** $G_{x}$ Channel, **B** $G_{y}$ Channel, **C** $G_{z}$ Channel. The third column depicts the relative difference between measured and simulated data as error metric. The red circles represent the spherical region of interest with a diameter of 3*mm*. Relative differences between measured and simulated are found to be below $8\%$ for all the $G_{x}$ and $G_{y}$ channel and below $6\%$ for the $G_{z}$ channel

Concerning the influence of the surface normal orientation $\vec {n}=\vec {n}(\theta ,\phi )$ of the bi-planar geometry, we examine various azimuthal ($0^{\circ }<\phi <360^{\circ }$) and polar angles ($0^{\circ }<\theta <90^{\circ }$) on the upper hemisphere, defined according to the *ISO 80000-2:2019* coordinate convention. The two other bi-planar parameters are set constant to $l=5cm$ and $d=3cm$. Normal vectors pointing to the lower hemisphere are excluded for symmetry reasons, as they would correspond to reversing the gradient direction by altering the current polarity. For each gradient channel ($G_{x}$, $G_{y}$, $G_{z}$), 102 evenly distributed normal orientations are studied over the hemispheres (See Fig. 1 and 3).

To ensure that different effects are not masked while studying the geometric parameters *l* and *d*, we fixed the azimuthal and polar angles of the surface normal $\vec {n}$ at $90^{\circ }$ and $35^{\circ }$, respectively, corresponding to the orientation depicted in Fig. 1b. For each set of design parameters, a set of coil layouts for the three channels is generated using the *CoilGen* software, which allows for a grid search within a specified parameter space. Each solution is evaluated for gradient sensitivity using the following equation:

1 $$\eta _{{xyz}} = \mathop {mean}\limits_{{Roi}} \left( {\frac{{\partial B_{z} (\vec{r})}}{{\partial {xyz} }}} \right)/I$$

To quantify the relative field error, we define the deviation from the linear target [30] as follows:

2 $$\begin{aligned} Err_\textrm{rel}(\vec {r}) = \frac{ |B_{z}(\vec {r})_\textrm{Layout}-B_{z}(\vec {r})_\textrm{Target} |}{ \mathrm max( |B_{z}\left( \vec {r})_\textrm{Target} |\right) } \end{aligned}$$

Equation  1 provides the sensitivity of the three channels ($G_{x}$, $G_{y}$, $G_{z}$) with respect to the resulting gradient field per unit current, while Eq.  defines the deviation from the target in terms of the magnetic field without differentiation. This approach allows for a more direct comparison with experimental field measurements.

### Generating the coil layouts

For each specified surface geometry (i.e., a set of values for the bi-planar parameters *l*, *d*, and $\vec {n}$), a coil layout is generated. The process is outlined as follows: Using the CoilGen software, a triangulated bi-planar surface mesh is generated based on the given values of *l*, *d*, and $\vec {n}$. This mesh represents the current-carrying surface, where an electric surface current density $\vec {j}$ is optimized to achieve the desired magnetic target field within the target region. The optimization is performed using the *stream function* approach [30, 31], which is implemented in the CoilGen software.

In the stream function approach, a surface current density $\vec {j}$ is defined and optimized based on a scalar stream function $\Psi$, where $\vec {j}=\vec {n}\times \vec {\nabla }\Psi$ [32, 33]. The stream function $\Psi$ is defined on the nodes of the current-carrying surface and determines the current density $\vec {j}$ that generates the desired magnetic field $\vec {B}$. In the context of MR, this is usually a spatial modulation of the z-component parallel to the static main field, $B_{0}$ [16]. In our case, the current-carrying surface is the bi-planar surface that we are optimizing in this study. The optimization problem for $\Psi$ and its explicit *Tikhonov* solution, as described by Poole et al. [34] and Calvetti et al. [35], is given by:

3 $$\begin{aligned} {\begin{matrix} \min _{\Psi } : || {\textbf {S}}\Psi -{\textbf {B}}_\textrm{Target} ||^{2}+ ||\lambda {\textbf {R}}\Psi ||^{2} \\ \Psi _\textrm{Opt}=({\textbf {S}}'{} {\textbf {S}} + \lambda ^{2}{} {\textbf {R}}'{} {\textbf {R}})^{-1}{} {\textbf {S}}'{} {\textbf {B}}_\textrm{Target} \end{matrix}} \end{aligned}$$

In this equation, ${\textbf {S}}$ represents the sensitivity matrix that relates the circular nodal currents to the target field coordinates based on Biot–Savart’s law. The matrix ${\textbf {S}}$ depends on the current-carrying surface geometry and needs to be recalculated for each bi-planar configuration. The expression ${\textbf {S}}\Psi$ gives the magnetic field generated by $\Psi$ at the target location. ${\textbf {R}}$ is the regularization matrix representing the electric resistance. The regularization parameter $\lambda$ balances field accuracy and power dissipation. A larger $\lambda$ increases sensitivity (gradient field per unit current) but may result in a higher field deviation from the linear target field (field error).

The optimized stream function result must be discretized into *n* equally spaced potential steps to obtain the wire turns of the coil, known as isocontour lines [33]. The number of potential steps *n* affects efficiency and field accuracy, with higher values improving accuracy but also increasing inductance and reducing the distance between coil windings. The CoilGen software is used to connect the turns and generate a 2D layout, which is saved as a vector graphic file (*.svg*) for manufacturing the coil through printed circuit boards (*PCBs*).

### Setup for the experimental validation

The wire pattern was implemented using two double-layer PCBs for each gradient channel, with a copper thickness of $35 \mu m$ (Beta LAYOUT GmbH, Aarbergen, Germany). The coil design’s performance was experimentally validated in a clinical 3T whole-body MRI scanner (MAGNETOM Prisma, Siemens Healthcare GmbH, Erlangen, Germany). To align the $B_{0}$ direction of the MRI scanner with the internally required $B_0$ orientation direction of the gradient system, a tilted PCB coil holder for the PCBs was designed using Blender v.2.7 software (Stichting Blender Foundation) and 3D printed using Polylactide on a Prusa i3 MK3S+ 3D printer (Prusa Research a.s).

An MRI phantom consisting of a small plastic bottle filled with copper sulfate solution was placed inside the gradient system. A $(7 \mathrm cm)$ loop coil was positioned on top of the setup for MR signal reception. Phase images were acquired using a double gradient echo sequence with echo times of $4 \mathrm ms$ and $7.72 \mathrm ms$. Phase differences were converted to corresponding field strengths using the equation $B=\gamma \cdot \Delta \Phi / \Delta TE$ [36]. The MR sequence parameters were chosen to achieve an isotropic resolution of $1mm$, with an echo spacing small enough to avoid phase wrapping artifacts.

Theoretical field maps were calculated from simulated wire tracks using Biot–Savart’s law.

## Results

More than 500 combinations of the bi-planar geometric parameters, including square side length (*l*), separation (*d*), and orientation ($\vec {n}$), were generated and analyzed in terms of sensitivity and deviation from the targeted field (Figs. 2, 3 and 4). During the stream function optimization, the regularization factor ($\lambda$) was set to a constant value of $\lambda = 100,000$ to account for power dissipation specific to the 2178 mesh nodes of the bi-planar current-carrying surface. This value was intentionally set higher than the optimal value determined by the L-curve [35] to further enhance sensitivity.

The influence of surface separation (*d*) and square side length (*l*) on gradient sensitivity and relative error was examined for the $G_{x}$ channel (Figs. 2). With a fixed square side length of $l = 10$ cm, sensitivity decreased as the separation increased, and significant field error occurred for separations larger than $d = 3$ cm.

Regarding the bi-planar square side length (*l*) with a fixed separation of $d = 3$ cm, sensitivity increased with larger surface sizes, but no further gain was observed for square side lengths greater than 5 cm. Significant field error occurred for surfaces with $l > 5$ cm.

The influence of surface orientation was investigated for all three channels by analyzing solutions with different polar angles ($\theta$) and azimuthal angles ($\phi$) on the upper hemisphere (Figs. 3, 4). For the transverse gradient channels $G_{x}$ and $G_{y}$, tilted bi-planar orientations exhibited higher efficiency compared to horizontal or vertical alignments. The highest sensitivity was achieved with a polar angle of $55^\circ$ in the direction of the respective gradient axis (Figs. 3, 4). For the $G_{z}$ channel, the highest sensitivity of approximately $25mT/m/A$ was obtained when the solution was directed towards the *z* axis.

Various optimization approaches were identified to enhance the overall performance of the gradient system (Fig. 4). One option is to balance the sensitivity of the $G_{z}$ channel with one of the transverse $G_{x}$ and $G_{y}$ channels, achieved by setting $\theta \approx 30^\circ$ and $\phi = 0^\circ$ or $\phi = 90^\circ$. Another possibility is to select $\theta \approx 40^\circ$ and $\phi = 45^\circ$, which balances the power of all channels but slightly reduces total sensitivity and symmetry of the $G_{x}$ and $G_{y}$ channels.

Despite the maximum polar angle of $55^\circ$ for either the $G_{x}$ or $G_{y}$ channel, the configuration with $\theta = 35^\circ$ and an azimuthal angle of $\phi = 90^\circ$ (Fig. 1b) was chosen for implementing the gradient coil set on PCBs. This orientation allows for an upright, horizontal alignment in the laboratory coordinate system, simplifying integration into the overall measurement setup.

The separations of the biplanar coils used for the different channels were adjusted to compensate for sensitivity differences. The $G_{x}$ channel, with the lowest sensitivity, was placed at the innermost position of the three-channel gradient system (Fig. 5 A). By using double-layered tracks and a slightly lower number of coil turns for $G_{z}$ (Table 1), all sensitivities were adjusted to a similar range of $26-29$ mT/m/A.

**Table 1:** Coil parameters of the chosen design: average sensitivity $\mathrm [mT/m/A]$, resistance $\mathrm [Ohm]$, inductance $[\mu H]$, maximum field error $[\%]$, number winding turns

Coil parameters					
channel	Mean sensitivity $\mathrm [mT/m/A]$	DC resistance $\mathrm [Ohm]$	Inductance $\mathrm [\mu H]$	Max field error $\mathrm [\%]$	Iso-contour levels (see winding variable *n* [29])
$G_{x}$	$28.66 \pm 0.19$	2.04	3.76	8	14
$G_{y}$	$26.82 \pm 0.12$	1.87	4.56	8	14
$G_{z}$	$26.00 \pm 0.18$	2.14	4.63	6	10

The inductance was calculate with the Software *FastHenry2*[40]

A complete gradient set combining $G_{x}$, $G_{y}$, and $G_{z}$ channels, with a square side length of $l = 5$ cm, bi-planar surface separations of $d = (3, 3.35, 3.7)$ cm, and an orientation of polar angle $35^\circ$ and azimuthal angle $90^\circ$, was manufactured using the layouts shown in Fig. 5 on double-layer PCBs (also see the prototype in Fig. 6). The tracks of the individual coils had a width of 0.5 mm and a minimal gap of 0.01 mm between them. The copper thickness of the PCBs was $35\mu$. All interconnections were designed to allow for a spiral back-and-forth connection without additional feed lines (Fig. 5).

Experimental and theoretical field maps are depicted in Fig. 7. The measured performance for the transverse $G_{x}$ and $G_{y}$ coils was 28.7mT/m/A and 26.8 mT/m/A, respectively, while the $G_{z}$ coil achieved 26 mT/m/A. The relative differences between measured and simulated values were below $8\%$ for $G_{x}$ and $G_{y}$, and below $6\%$ for $G_{z}$ (Fig. 7).

## Discussion

The aim of this study was to design an optimal bi-planar gradient configuration for the diffusion-weighting experimental NV-NMR setup. We successfully implemented a three-channel gradient coil set on PCBs. However, this work can also be considered a more general study of bi-planar geometric parameters and their influence on gradient performance.

In terms of parameter selection for the final built system, we chose a square side length of $l=5 \mathrm cm$ due to limited available space and the absence of any observed advantage in sensitivity or accuracy in the parameter study (see Fig. 2C). The saturation of sensitivity for increasing *l* can be attributed to two factors. First, the surface area appears sufficient as fewer windings are placed on radially outer locations. Second, without dynamic adjustment of node density, the mesh resolution decreases with increasing *l*, especially for the relevant inner surface region.

For the bi-planar surface separation *d*, we found that sensitivity is inversely related (see Fig. 2A). We chose a separation of $d=3 \mathrm cm$ as the smallest value that avoids interfering with the optical path of the existing NV–NMR setup and overlapping with the RF excitation coils. However, this parameter had the strongest influence on sensitivity. Future designs should position the gradient coils as close as possible to the target region.

Regarding the influence of surface orientation $\vec {n}$, we notably found that polarly tilted bi-planar orientations have higher efficiency for the transverse gradient channels than horizontal or vertical orientations. As seen from the plots in Figs. 3 and 4, we evaluated two options for implementing a gradient system with balanced near-optimal performance. Option 1 involves choosing a polar angle of $\theta =30^{\circ }$ and an azimuth of $\phi =0^{\circ }$ (or $\phi =90^{\circ }$), balancing the performance of one of the transverse ($G_{x}$ or $G_{y}$) coils with the $G_{z}$ gradient while accepting a lower performance of the remaining channel. Option 2 involves balancing the sensitivities of all three axes with an azimuth of $\phi =45^{\circ }$, resulting in a homogeneously reduced performance. We chose a solution close to option 1 with $\theta =35^{\circ }$ because we could partially compensate for the sensitivity differences with the final ordering of the coils within the overall system, which automatically implies different bi-planar surface separations (in our case, $3 \mathrm cm, 3.35 \mathrm cm$, and $3.75 \mathrm cm$). Furthermore, the polar angle of $\theta = 35^{\circ }$ is required by the NV-NMR setup for a simple straight horizontal integration of the bi-planar gradient system in the laboratory coordinate frame (see Fig. 1b–d). Coincidentally, the optimal transversal polar angle $\theta =55^{\circ }$ is not far from the advantageous bi-planar angle of $\theta = 35^{\circ }$ for the NV-NMR setup.

We do not claim to have found a universally optimal polar angle for transversal bi-planar gradients. The optimal polar angle also depends on other geometric parameters such as surface separation and design variables like the regularization factor $\lambda$. The optimal polar angle $\theta$ must be found specifically for each problem. However, it has consistently been shown that inclined bi-planar alignments achieve higher efficiencies for transverse gradient coils.

The relative orientation of the gradient field axes relative to $B_{0}$ and the NV axis was not optimized to avoid further increasing the parameter space and computational effort. In general, gradient directions do not necessarily have to coincide with the respective *x*, *y*, and *z* axes of the internal coordinate system, which in our case is the coordinate system of the diamond NV center. Therefore, further improvements and design variants in this respect are possible in the future.

With a gradient sensitivity of over $26 \mathrm mT/m/A$ for all three channels in the built system, it is possible to obtain a gradient strength of 100*mT*/*m* with a current of only 3.8*A*. Such high gradient strengths are desirable for obtaining high b-values for diffusion-based NV–NMR experiments. However, in DC mode, 3.8*A* would lead to intolerable heating of the PCBs. With a copper thickness of $35\mu m$ for the tracks and an average width of 0.5*mm*, the cross-section can safely support only a constant DC strength of 1*A* without significant heating above an additional $10^{\circ }C$ [37]. Nevertheless, since the current duty cycle of diffusion weighting pulses is typically less than $10\%$ [38], it will be possible to apply pulsed currents with higher current strength without exceeding the thermal loading of the system. However, future versions of the coils should use thicker tracks to further reduce resistance heating.

With the relatively small values of $3-5 \mu H$ for the magnetic inductance (see Table 1), no peak voltage limitations are expected while driving the coils, unlike whole-body MR gradient systems. Therefore, as typical for small gradient coils [39], the performance of the presented system is limited by ohmic losses rather than inductance.

To the best of our knowledge, no detailed studies analyzing the effects of varying side lengths, surface separations, and orientations of bi-planar current-carrying surfaces have been conducted. We hope that this study helps foster more efficient gradient coil designs in the future, which may include further optimization parameters such as the size and shape of the target area (or field of view), active shielding, and optimizing the relative orientation between the gradient directions and the respective coordinate axes.

## Conclusion

A bi-planar MR gradient system was successfully designed, implemented, and experimentally validated for NV-NMR-based experiments. Through an extensive exploration of over 500 layout designs incorporating varying square side lengths, surface separations, and orientations, we determined that inclined orientations offer higher efficiency for the $G_{x}$ and $G_{y}$ channels compared to vertical or horizontal orientations. Taking these findings into consideration, we selected a design with a $35^{\circ }$ inclination and implemented a three-channel gradient system on PCBs. Sensitivity measurements revealed values of 28.7*mT*/*m*/*A* and 26.8*mT*/*m*/*A* for the transverse $G_{x}$ and $G_{y}$ gradients, respectively, while the $G_{z}$ gradient exhibited a sensitivity of 26*mT*/*m*/*A* within a spherical target region with a diameter of 3*mm*. Notably, the measured results aligned well with the predicted magnetic fields for all three channels, confirming the effectiveness of the bi-planar gradient system.
